# Repair methods in peripheral nerves after traumatic injuries: a systematic literature review

**DOI:** 10.1590/acb401225

**Published:** 2025-02-24

**Authors:** Naely Nobre Costa, Jennifer Ferreira dos Santos, Maria Fernanda de Almeida Cavalcante Aranha, Erik William Farias Coelho, Victor Leno Silva Paes, Rita de Cássia Silva de Oliveira

**Affiliations:** 1 Universidade do Estado do Pará – Medical School – Belém (PA) – Brazil.

**Keywords:** Peripheral Nerve Injuries, Plastic Surgery Procedures, Systematic Review

## Abstract

**Purpose:**

To identify and describe the most used surgical repair methods for traumatic injuries to peripheral nerves, as well as highlight the causes of trauma to peripheral nerves and the most prevalent traumatized nerves.

**Methods:**

This is a systematic literature review using the recommendations of the Preferred Reporting Items for Systematic Reviews and Meta-Analyses (PRISMA). The searches were carried out in PubMED, in the time window from January 2018 to December 2022.

**Results:**

In total, 3,687 articles were collected, of which, after applying the inclusion and exclusion filters and analyzing the risk of bias, 34 articles remained. It was observed that the age of the injury and type of nerve repair strongly influence the recovery of patients. The most identified trauma repair procedures were neurolysis, direct suturing, grafting, and nerve transfer. Among these four procedures, direct suturing is currently preferred.

**Conclusion:**

Several repair methods can be used in peripheral nerve injuries, with emphasis on direct suturing. However, nerve transfer proves to be a differential in those cases in which repair is delayed or the first treatment options have failed, which shows that each method will be used according to the indication for each case.

## Introduction

Peripheral nerve injuries directly affect the quality of life of affected patients, as they reduce functional capacity and physical independence. Physiopathologically, these injuries are represented by various alterations in the nervous tissue: increased metabolism in the cell body, mainly of Schwann cells, exhibiting nuclear and cytoplasmic increase, as well as an increase in the mitotic rate to promote repair, degeneration of the segment proximal and distal to the lesion through the removal of axonal debris and degenerated myelin, regeneration of the myelin sheath, which will not be restored to the same quality as before, and regeneration of the axon itself. However, these regeneration processes only occur if the cell body, located in the anterior horn of the spinal cord or in paravertebral ganglion, is preserved^1^.

Regarding the mechanisms of nerve injuries, the Seddon classification is important in stratification. The first type is neuropraxia, considered the least serious. It is associated with compressive trauma, with local damage to myelin and sensory and motor loss, but without compromising the axon. The second variation is axonotmesis, caused by crushing or stretching and assessed as more serious than neuropraxia, as it involves damage to the myelin and axon, but regeneration is still possible. The third type is represented by neurotmesis, classified as the most serious of the three, in which the nerve completely ruptures due to a direct traumatic or iatrogenic impact (cut or pressure), preventing axonal growth, and surgery as soon as possible is the only effective form of repair^2^^,^ 3.

Regarding treatment, it may vary depending on the type of injury. Therefore, open injuries, associated with complete or partial sections of the nerves (neurotmesis), must be treated surgically as quickly as possible. In closed injuries, which preserve the continuity of the nerve (neuropraxia and axonotmesis), it is recommended to wait three months before performing surgery, as during this time there is the possibility of axonal regeneration. In cases in which there is no possibility of regeneration, three types of surgical repair can be used: epineural repair, perineural or fascicular repair, repair between groups of fascicles, grafts, and conductive tubes, and they will be chosen according to each case^1^.

It is possible to identify that workplaces are susceptible scenarios for injuries that can compromise the motor functions of the affected limb; that, when they are severe, they tend to be caused by sharp objects and are proximal injuries, generally characterized as a disruptive injury both to the axon and its myelin coating and to the integrity of the perineurium^4^. However, it is possible to observe the lack of epidemiological studies analyzing the incidence of peripheral nerve injuries in the country. Around the world, other countries also reveal data in which men are the most affected by injuries to peripheral nerves, such as Sweden in an epidemiological study, in which manual labor positions were the main cause of these injuries^5^.

Given this entire panorama, it is necessary to clarify that some factors directly alter the prognosis of patients who have undergone surgical procedures for injuries to peripheral nerves, such as: age, type of wound, nerve repair, extent of the injury, and the period between the accident and the treatment received, some of which will be discussed in this study. That said, the objectives of this work were to identify and describe the most used surgical repair methods for traumatic injuries to peripheral nerves, as well as highlight the causes of trauma to peripheral nerves and the most prevalent traumatized nerves.

## Methods

### Type of study, information sources and registration

The study is a literature review whose guiding question is: what are the main methods of repairing peripheral nerves that have suffered traumatic injuries? In this sense, the recommendations of the Preferred Reporting Items for Systematic Reviews and Meta-analyses (PRISMA) was used, and the searches were carried out in the Medical Literature Analysis and Retrieval System Online (MEDLINE) in its PubMED search engine, using the RAYYAN software to organize the reading and selection of articles by the researchers. The study was registered on the OSF Registries platform under registration number 10.17605/OSF.IO/C32W7.

### Search strategy

Regarding descriptors and Boolean operators, the following were employed: Repair Neurosurgery; Repair Neurological Surgery; Repair AND Endonerium; Repair AND Perineurium; Repair AND Peripheral Nerve; Peripheral Nerve Injury OR Peripheral Nerve Damage; Nerve injury; Traumatic Peripheral Nerve Injury; Peripheral Nerve Repair Surgery; Nerve Repair Methods.

### Eligibility criteria, selection, and data collection process

By initially using Rayyan software to read titles and abstracts and then reading the articles in full by the reviewers themselves, articles and journals published in the time window from January 2018 to December 2022 were included, which dealt with peripheral nerve repair techniques after trauma, available in full and in Portuguese, English and Spanish. In addition, systematic reviews, studies carried out on cadavers, case reports, studies carried out on animals and studies of injuries caused by iatrogenic injuries were excluded. The researchers carried out the review processes individually, following the eligibility criteria.

### Data items and study risk of bias assessment

A list of selected primary studies was created, and, to analyze the risk of bias, some tools were used based on the type of study. For randomized clinical trials, Risk of Bias 2.0 (Rob 2.0) was used. For non-randomized clinical trials, the Risk of Bias in Non-Randomized Studies of Interventions-1 (ROBINS-1) was used.

## Results and Discussion

**Figure 1:**
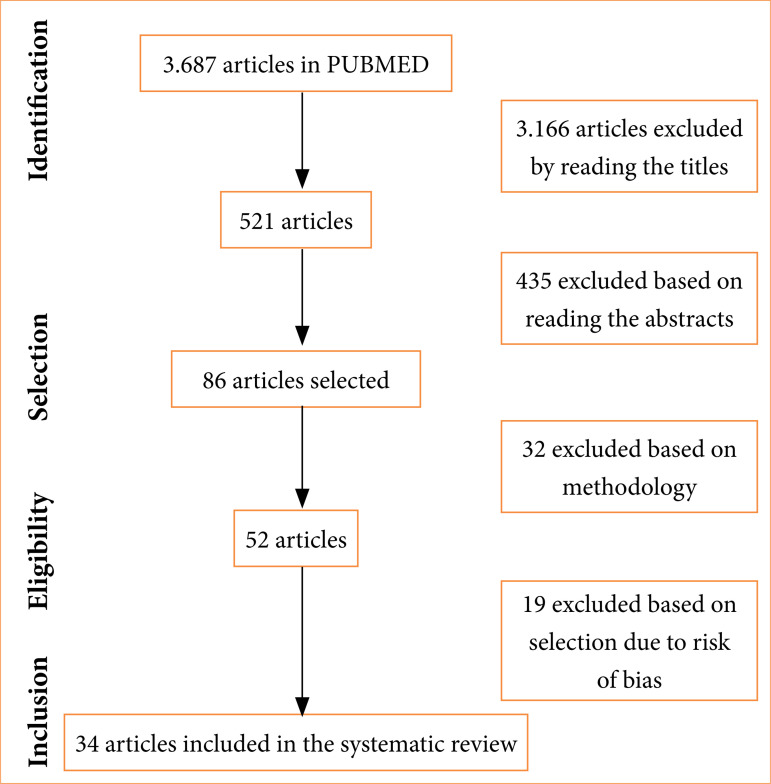
Preferred Reporting Items for Systematic Reviews and Meta-analyses flowchart.

Source: Elaborated by the authors.

Figure 1 reveals the PRISMA flowchart, following the inclusion and exclusion criteria for selecting studies. In total, 3,687 articles were collected, of which, after reading the titles, abstracts, and methodological process, 52 articles remained, and, among these, after analyzing the risk of bias, 34 articles remained and were included in this review as they presented a low risk of bias.

Overall, the risk of bias of the randomized and non-randomized studies in this systematic review was low. Therefore, in this case, the analyses and conclusions obtained could be qualified. However, it is worth noticing that 18 non-randomized studies presented a risk of bias in domain 7. Thus, these studies may raise concerns about obtaining significant conclusions due to bias found in the selection of reported results (Tables 1 and 2).

**Table 1:** Risk of bias analysis for included randomized clinical trials.

Authors	D1	D2	D3	D4	D5	General
Neubrech et al. (2018)^6^	**+**	**+**	**–**	**+**	**+**	**+**
Sallam et al. (2022)^7^	**+**	**+**	**+**	**+**	**+**	**+**
Shahraki et al. (2022)^8^	**+**	**+**	**+**	**+**	**+**	**+**

D1: bias arising from the randomization process; D2: bias due to deviations from the intended intervention; D3: bias due to lack of outcome data; D4: bias in outcome measurement; D5: bias in the selection of the reported result; **–**Moderate; **+**Low. Source: Elaborated by the authors.

**Table 2:** Risk of bias analysis for the non-randomized studies included.

Authors	D1	D2	D3	D4	D5	D6	D7	General
Bulstra et al. (2018)^9^	**+**	**+**	**+**	**+**	**+**	**+**	**+**	**+**
Smith et al. (2018)^10^	**+**	**–**	**–**	**–**	**–**	**+**	**+**	**–**
Liu et al. (2018)^11^	**+**	**–**	**+**	**+**	**+**	**+**	**+**	**+**
Yang et al. (2018)^12^	**+**	**–**	**+**	**+**	**+**	**+**	**+**	**+**
Rasulic et al. (2018)^13^	**–**	**–**	**–**	**–**	**–**	**–**	**–**	**–**
Siqueira et al. (2019)^14^	**+**	**+**	**+**	**+**	**–**	**+**	**–**	**+**
Siqueira et al. (2019)^15^	**+**	**–**	**+**	**+**	**–**	**+**	**–**	**+**
Xiao et al. (2019)^16^	**+**	**–**	**+**	**+**	**+**	**+**	**–**	**+**
Shore et al. (2019)^17^	**+**	**+**	**+**	**+**	**–**	**+**	**–**	**+**
Li et al. (2019)^18^	**+**	**–**	**+**	**+**	**+**	**+**	**–**	**+**
Degeorge et al. (2019)^19^	**+**	**+**	**+**	**+**	**–**	**+**	**+**	**+**
Karabeg et al. (2019)^20^	**+**	**+**	**+**	**+**	**–**	**+**	**–**	**+**
Rasulic et al. (2019)^21^	**–**	**–**	**+**	**+**	**+**	**+**	**–**	**+**
Monsivais (2020)^22^	**+**	**+**	**+**	**+**	**–**	**+**	**+**	**+**
Cao et al. (2020)^23^	**+**	**+**	**+**	**–**	**+**	**+**	**+**	**+**
Emamhadi et al. (2020)^24^	**+**	**–**	**+**	**+**	**+**	**+**	**–**	**+**
Socolovsky et al. (2020)^25^	**+**	**–**	**+**	**+**	**+**	**–**	**–**	**+**
Pages et al. (2020)^26^	**+**	**+**	**+**	**–**	**–**	**+**	**+**	**+**
Solla et al. (2022)^27^	**+**	**+**	**–**	**+**	**+**	**–**	**–**	**+**
Leversedge et al. (2020)^28^	**+**	**+**	**–**	**+**	**+**	**+**	**+**	**+**
Smith et al. (2020)^29^	**+**	**–**	**–**	**+**	**+**	**+**	**–**	**+**
Souza et al. (2020)^30^	**+**	**+**	**–**	**–**	**–**	**+**	**–**	**–**
Emamhadi et al. (2021)^31^	**+**	**+**	**+**	**+**	**+**	**+**	**+**	**+**
Wang et al. (2021)^32^	**+**	**+**	**+**	**+**	**–**	**+**	**–**	**+**
Dunn et al. (2021)^33^	**–**	**+**	**–**	**–**	**+**	**–**	**+**	**–**
Tsymbaliuk et al. (2021)^34^	**–**	**–**	**+**	**–**	**+**	**–**	**–**	**–**
Lordache et al. (2021)^35^	**–**	**+**	**+**	**–**	**–**	**–**	**–**	**–**
Temiz et al. (2021)^36^	**–**	**+**	**+**	**–**	**+**	**–**	**–**	**–**
Rasulic et al. (2021)^37^	**+**	**+**	**+**	**+**	**–**	**–**	**+**	**+**
Zhan et al. (2022)^38^	**+**	**+**	**+**	**+**	**+**	**+**	**+**	**+**
Garutti et al. (2023)^39^	**–**	**+**	**+**	**–**	**–**	**–**	**–**	**–**

D1: Bias due to confusion; D2: Bias due to participant selection; D3: Bias in the classification of interventions; D4: Bias due to deviations from intended interventions; D5: Bias due to missing data; D6: Bias in measuring results; D7: Bias in the selection of the reported result; **–**Moderate; **+**Low. Source: Elaborated by the authors.

Some variables relating to the specialty and type of injury were identified based on the analysis of the 34 articles. Regarding specialty, the most prevalent was neurosurgery, present in 16 articles, followed by traumatology and orthopedics, with eight articles, hand surgery with six articles, and plastic surgery in four articles.

Based on the analysis of the types of injuries, injuries were identified from automobile accidents, falls, industrial accidents, war injuries, spinal cord injuries, accidents during sports practice, crushing, perforation, laceration, firearm injuries, and brachial plexus injuries resulting from of labor. Table 3 summarizes the primary articles included in this research.

**Table 3:** Results based on reading the articles.

Authors	Sample number	Age	Method	Injured nerve	Nerve used
Neubrech et al. (2018)^6^	74	Average of 43 years old	Placement of a chitosan nerve tube on the injured nerve	Unspecified sensory nerve of the fingers	Does not fit
Sallam et al. (2022)^7^	85	20 to 58 years old	Microsuture with epineural surgical repair and nerve repair with autologous fibrin glue	Ulnar nerve and median nerve	Does not fit
Shahraki et al. (2022)^8^	30	Average of 35 years old	Suture neurorrhaphy, with amniotic membrane coverage around the nerve	Peripheral nerves	Does not fit
Bulstra et al. (2018)^9^	42	Average of 27.7 years old	Transfer of the spinal accessory nerve to the motor branch of the radial nerve	Brachial plexus: motor branch of the radial nerve	Spinal accessory nerve
Smith et al. (2018)^10^	13	Average of 7.4 months old	Nerve transfer from the musculocutaneous nerve to the ulnar nerve fascicle	Musculocutaneous nerve	Ulnar fascicle
Liu et al. (2018)^11^	21	17 to 51 years old	Phrenic nerve or ulnar nerve transfer to the brachial plexus	Brachial plexus	Phrenic nerve or ulnar nerve
Yang et al. (2018)^12^	95	18 to 58 years old	Nerve transfer	Brachial plexus: median nerve	Ulnar nerve
Rasulic et al. (2018)^13^	60	16 to 52 years old	Nerve grafting, split repair, interfascicular neurolysis, external neurolysis and exploration, as well as nerve transfer in the musculocutaneous and axillary nerve	Spinal nerves C5, C6, C7, C8, and T1	Does not fit
Siqueira et al. (2019)^14^	104	0 to 2 years old	Nerve transfer, external neurolysis, nerve grafting or combination of techniques	Injury to C5 and C6 (group 1); C5, C6, and C7 (group 2); C5 to T1 (group 3) and C5 to T1 with associated Horner syndrome (group 4)	Does not fit
Siqueira et al. (2019)^15^	76	Upper plexus injuries: average of 24.6 years old; complete brachial plexus palsy: 26.2 years old	Suprascapular nerve repair	Suprascapular nerve	Spinal accessory nerve
Xiao et al. (2019)^16^	20	Average of 28.9 years old	Transfer of the spinal accessory nerve to the suprascapular nerve; transfer of the nerve branch from the long head of the triceps to the axillary nerve and transfer of two intercostal nerves to the axillary nerve	C5-C6; C5-C7	Spinal accessory nerve-suprascapular nerve and spinal accessory nerve-suprascapular nerve together with intercostal nerve or long branch of triceps nerve-axillary nerve
Shore et al. (2019)^17^	243	Average of 6.7 years old	Neurolysis, median nerve decompression, median nerve sural cable graft, radial nerve decompression and tendon transfer	Median, radial, ulnar nerve, or combination of nerve injuries	Does not fit
Li et al. (2019)^18^	16	Average of 27.9 years old	Nerve transfer	Brachial plexus	ulnar nerve
Degeorge et al. (2019)^19^	58	Average of 24 years old	Suprascapular nerve	Suprascapular nerve	Accessory spinal nerve
Karabeg et al. (2019)^20^	40	FCR Group: average age of 37.95 years old; FCU group: average age 38.75 years old	Transfer of tendons innervated by the ulnar or median nerve to the tendon paralyzed by the radial nerve injury, using the flexor carpi ulnaris tendon (FCU) or flexor carpi radialis tendon (FCR)	Radial nerve	Ulnar nerve and median nerve
Rasulic et al. (2019)^21^	36	Average of 21 years old	Use of the proximal stump of the C5 spinal nerve for grafting into the musculocutaneous nerve and axillary nerve. In some cases, the proximal stump of the C6 spinal nerve is used to graft the medial radial and pectoral nerve	Brachial plexus	C5 spinal nerve and C6 spinal nerve
Monsivais (2020)^22^	11	5 to 47 years old	Contralateral C7 transfer, standard C5 free graft, and spinal attachment transfer	Median nerve, suprascapular, cutaneous muscle, intercostals T4, T5 and T6	C7, C5 and accessory nerve
Cao et al. (2020)^23^	9	Not reported	Nerve graft, obturator nerve trunk transfer and transfer of distal mortal branches of the obturator	Femoral nerve	Obturator nerve
Emamhadi et al. (2020)^24^	7	14 to 56 years old	Transfer of the motor fascicle of the ulnar nerve to the long head of the triceps branch of the radial nerve	Motor fascicle of the ulnar nerve	Radial nerve
Socolovsky et al. (2020)^25^	131	16 to 35 years old	Phrenic nerve or musculocutaneous nerve	Musculocutaneous nerve	Phrenic nerve and spinal accessory nerve
Pages et al. (2020)^26^	29	21 to 59 years old	Nerve root grafting, direct transfer, graft transfer	C5/C6	Musculocutaneous, supraspinal and axillary nerve
Solla et al. (2022)^27^	83	9 to 28years old	Spinal accessory transfer to suprascapular nerve	Suprascapular nerve	Spinal accessory nerve
Leversedge et al. (2020)^28^	110	16 to 45 years old	Processed nerve allograft and conduits	Sensory digital nerve	Does not fit
Smith et al. (2020)^29^	45	7 months old in average	Spinal accessory nerve transfer to suprascapularis and graft repair	C5 - T1	C5 or C6 and spinal accessory nerve
Souza et al. (2020)^30^	11	24 to 59 years old	Transfer of the motor branch of the brachialis to the anterior interosseous nerve and the supinator branch to the posterior interosseous nerve in a first surgical procedure and transfer of the anterior interosseous nerve to the pronator quadratus branch of the ulnar nerve	Anterior and posterior interosseous nerve	Brachial nerve
Emamhadi et al. (2021)^31^	39	Average of 31 years old	Transfer of the motor fascicle from the ulnar carpal branch of the ulnar nerve was used in the mid-superior brachial plexus, as well as transfer of the intercostal nerves to the biceps branch of the musculocutaneous nerve in the total superior brachial plexus	Cutaneous muscle nerve	Intercostal nerves and ulnar nerve
Wang et al. (2021)^32^	27	Average of 33 years old	The lower trunk was mobilized by separating it from the posterior division and the medial cutaneous nerve of the forearm distally. The mobilized lower trunk was then directly approached to an ipsilateral root stump	C5, C6, C7, C8, and T1	Does not fit
Dunn et al. (2021)^33^	23	21 to 49 years old	Processed nerve allograft (Avance Nerve Graft; AxoGen, Inc., Alachua, FL)	Sciatic, lateral femoral cutaneous nerve	Does not fit
Tsymbaliuk et al. (2021)^34^	138	18 to 62 years old	Nerve decompression, nerve suture and nerve grafting using sural nerve	Sciatic, ulnar, median, radial, tibial, common peroneal	Sural nerve in graft cases
Lordache et al. (2021)^35^	44	7 to 52 years old	Nerve transfer and sural nerve graft	Brachial plexus, ulnar nerve, median, radial, sciatic nerve	Sural nerve in graft cases
Temiz et al. (2021)^36^	182	3 to 67 years old	Neurolysis, epineural anastomosis and sural nerve graft	Sciatic, peroneal nerve	Sural nerve in graft cases
Rasulic et al. (2021)^37^	77	12 to 75 years old	External neurolysis, longitudinal epineurotomy, epineurotomy circumferential and interfascicular neurolysis, direct nerve suture, graft	Radial nerve	Not reported
Zhan et al. (2022)^38^	29	18 to 61 years old	Neurolysis	Lumbosacral plexus injury	Does not fit
Garutti et al. (2023)^39^	36	Average of 47.5 years old	Direct nerve suture, direct nerve suture reinforced with fibrin glue, direct nerve suture with venous involvement	Lateral radial and ulnar digital nerve, common digital nerve	Does not fit

Source: Elaborated by the authors.

The study found that motor vehicle accidents, mainly motorcycles, were the main causes of peripheral nerve trauma, with accidents involving cars and pedestrians being observed^9^^,^ 12 ^,^ 18 ^,^ 26 ^,^ 27 ^,^ 31 ^,^ 32 ^,^ 34 ^,^ 35 ^,^ 37 ^,^ 38. Regarding brachial plexus avulsion, similar results were observed, that is, motorbikes were the most prevalent causes of peripheral nerve trauma^9^^,^ 18 ^,^ 27 ^,^ 40.

Another prominent etiology of peripheral nerve injuries is normal births, commonly affecting the brachial plexus^10^^,^ 14, which are caused by shoulder dystocia, fetal macrosomia, birth with forceps or other instrumentation, multiparity, prolonged labor and fetal malpresentation, with cesarean deliveries representing only 1% of cases^14^^,^ 29 ^,^ 41, hence the importance of carrying out prenatal care correctly to prevent this type of accident. Other causes were identified in studies: crushing, falling weight on the shoulder, earthquake, gunshots, sports, amputation, wars and falls from great heights, resulting from accidents, natural disasters, or violence^9^^,^ 12 ^,^ 13 ^,^ 18 ^,^ 21 ^,^ 26 ^,^ 28 ^,^ 31 ^–^ 38.

As for the time between the injury and surgical treatment, the studies emphasize that, in situations of covered nerve injury, spontaneous regeneration should be expected within the first three months, with a consensus that a period of less than six months is the best time for surgical treatment of this type of injury^13^^,^ 15 ^,^ 26. In the case of simultaneous nerve and vascular injuries, it was not possible to determine the time lapse between the injury and surgical correction from the articles included, since there are multiple causes that lead to different times for starting to treat the nerve injury, and the first intervention for these injuries does not always coincide with the start of treatment for nerve injuries^13^.

In relation to specialties, several areas of medicine that perform surgeries to repair traumatized peripheral nerves were identified: neurosurgery, orthopedic hand surgery, pediatric neurosurgery, plastic surgery, pediatric orthopedic surgery, orthopedic surgery, and orthopedics and traumatology^9^^,^ 6 ^,^ 12 ^,^ 17. However, it is noticed that neurosurgery is the most common specialty in these procedures, precisely because it is dedicated to repairing dysfunctions of the central and peripheral nervous system, with the aim of preserving as much as possible or restoring cognitive and executive functions^21^^,^ 24 ^,^ 25 ^,^ 27 ^,^ 29 ^,^ 30.

Another very obvious specialty is plastic surgery, possibly due to its restorative and aesthetic role, capable of improving the appearance of these injuries and restoring patients’ self-esteem^6^^,^ 11 ^,^ 20 ^,^ 23 ^,^ 41. From another perspective, primary articles were found involving surgeries in the field of orthopedics, as it is an area that usually deals with patients who have suffered traumatic injuries, using a range of technologies in their procedures, such as the prostheses^7^^,^ 8 ^,^ 12 ^,^ 17 ^,^ 19 ^,^ 22 ^,^ 26 ^,^ 32 ^–^ 35 ^,^ 38 ^,^ 39 ^,^ 42.

Despite the variety of surgical professionals capable of repairing injured peripheral nerves, all these surgeons have in common the ability and commitment to manipulate the extremely small and complex components of nerve structures using microsurgical techniques, regardless of the professional’s area of expertise. This understanding opens the possibility of using robotic surgery to repair peripheral nerve injuries^43^.

It is also worth noticing that another factor that can affect the prognosis of patients undergoing surgery to repair peripheral nerve lesions is age, since a correlation has been demonstrated between ageing and the capacity for nerve regeneration, given that, with advancing age, Schwann cells produce myelin more slowly, the main forms of anterograde axonal transport are delayed, and there is a deficit in the trophic responsiveness of injured neurons, as well as a decrease in macrophages in aged nerves, which can consequently delay regeneration even more^44^.

Fundamentally, three types of surgical procedures are used in the treatment of acute traumatic injuries to peripheral nerves: neurolysis, direct suturing, and nerve grafting^45^ Neurolysis consists of the resection of scar tissue from the nerve trunk and was identified in six studies evaluated by this review^13^^,^ 14 ^,^ 17 ^,^ 36 ^–^ 38. Neurolysis can be external, when cleaning is carried out around the epineurium, freeing it from adhesions to neighboring tissues, and internal or fascicular, when the epineurium is opened and scar tissue resection is carried out between the fascicles, with the aim to decompress the nervous trunk, groups of fascicles and, eventually, individual fascicles^38^.

The suture of a peripheral nerve aims exclusively to bring the endoneurium conduits closer together to facilitate the passage of fibers in regeneration through the injury in which there has been a solution to the continuity of the connective framework. It is the best way to repair an injury of a peripheral nerve^46^ and was identified in five studies in this review^7^^,^ 8 ^,^ 34 ^,^ 37 ^,^ 39.

The graft involves the use of a segment of nerve removed from a sensory nerve, generally the sural nerve, which will undergo a process of degeneration and will only function as a conduit for the axons in regeneration to reach the distal stump of the injured nerve^33^^,^ 47.

This type of procedure was identified in 13 articles in this review^13^^,^ 14 ^,^ 17 ^,^ 21 ^–^ 23 ^,^ 26 ^,^ 28 ^,^ 29 ^,^ 33 ^,^ 35 ^–^ 37. In the upper limb, the most used nerves are the medial cutaneous nerve of the forearm and the lateral cutaneous nerve of the forearm^45^. The sural nerve, in the lower limb, is considered the standard nerve graft and it is the most used due to the most suitable diameters and lengths (up to 30 cm in length)^48^^,^ 49. In neglected cases, it is recommended to repair the nerve within one year after the traum^a^ 50. The results with the use of grafts are not entirely satisfactory, as the number of nerves that go beyond the two suture lines varies between 37–50%, even though the use of grafts is considered the gold standard for late reconstructions, due to low morbidity. Vascularized grafts have been also described, in an attempt to reduce the endoneurial scar through less infiltration of fibroblasts^50^^,^ 51.

Nerve transfer surgery, also called neurotization, is generally indicated in cases of late presentation, failure of primary nerve reconstruction, isolated deficit, absence of proximal root for graft, and multiple avulsions of the nerve root^10^^,^ 20 ^,^ 26 ^,^ 29 ^,^ 31 ^,^ 52 ^,^ 53 ^,^ 54. This procedure was identified in 22 primary studies in this review^9^^–^ 20 ^,^ 22 ^–^ 27 ^,^ 29 ^–^ 31 ^,^ 35. In this procedure, branches of a neighboring nerve are removed and redirected to the distal end of the damaged nerve. After surgery, there are regeneration of the axons of the new pathway and reconnection of the motor cortex to relearn muscle functions^53^.

Strategies for repairing the brachial plexus consist of surgical exploration followed by reconstruction, using nerve grafts or nerve transfer^54^. Graft reconstruction is reserved only for post-ganglionic injuries. In preganglionic lesions (those in which root avulsion has occurred), the proximal stumps are not available for graft repair, and the surgical approach is based on nerve transfers^54^^–^ 56. Neurotization interneural can be intraplexual, between the nerves that originate from the brachial plexus, or extraplexual, when the donor nerves do not belong to the brachial plexus. Donors include the nerves for the long or lateral portion of the triceps brachii muscle, the intercostal nerves, the accessory nerve (XI cranial nerve), the phrenic nerve, the contralateral C7 root or the hypoglossal nerve (XII cranial nerve)^57^^,^ 58.

Methods other than the traditional nerve surgical suture technique are described in the literature of this review, such as the use of fibrin glue, which is a sealant composed of fibrinogen and thrombin. The use of this technique was associated with motor and sensory results similar to the use of the suture technique, although the use of fibrin glue was associated with shorter surgical time, so the choice to use this technique requires a cost-benefit analysis^7^^,^ 39.

Another method of repairing peripheral nerves is based on preventing perineural adhesions and scar formation in traumatic peripheral injuries using amniotic membrane wrapping. In the universe of application of this method, in terms of regeneration, the group that underwent the intervention achieved nerve regeneration and functional recovery of the nerve in a period of 12 months, while the control group did not experience nerve recovery and had functional and sensory impairment. It should be noted that this technique is one of the simplest ways of preventing neuroma at the site of injury and seems to prevent adhesions and perineural scarring^8^.

This systematic review has as limitations the heterogeneity of the sample number and the age of individuals included in the eligible studies, since it is difficult to obtain clinical trials randomized in interventions in the surgical area of peripheral nerve injuries due to the challenge of masking in these studies, which implies an analysis more restricted to non-randomized findings. Another important limitation is the fact that injured peripheral nerves are remarkably diverse, making it not possible to perform an analysis that includes all types of nerves. Despite these limitations, the conclusions obtained in this review provide direction for future research that aims to evaluate the variables associated with peripheral nerve injuries more specifically.

## Conclusion

The age of injury and type of nerve repair strongly influence patient recovery. The areas of medicine involved in the repair of peripheral nerves are neurosurgery, orthopedics, and plastic surgery, with emphasis on neurosurgery, which has proven to be the most active specialty in the repair of these injuries. These specialties include some trauma repair procedures: neurolysis, direct suturing, grafting, and nerve transfer. Direct suturing is currently preferred. However, nerve transfer proves to be a differentiator in those cases in which the repair is delayed, or the first treatment options have failed, which shows that each method will be used according to the indication for each case.

## Data Availability

All data sets were generated or analyzed in the current study.
